# An Effective Method to Purify *Plasmodium falciparum* DNA Directly from Clinical Blood Samples for Whole Genome High-Throughput Sequencing

**DOI:** 10.1371/journal.pone.0022213

**Published:** 2011-07-18

**Authors:** Sarah Auburn, Susana Campino, Taane G. Clark, Abdoulaye A. Djimde, Issaka Zongo, Robert Pinches, Magnus Manske, Valentina Mangano, Daniel Alcock, Elisa Anastasi, Gareth Maslen, Bronwyn MacInnis, Kirk Rockett, David Modiano, Christopher I. Newbold, Ogobara K. Doumbo, Jean Bosco Ouédraogo, Dominic P. Kwiatkowski

**Affiliations:** 1 Wellcome Trust Sanger Institute, Wellcome Trust Genome Campus, Hinxton, United Kingdom; 2 Global Health Division, Menzies School of Health Research, Charles Darwin University, Darwin, Australia; 3 London School of Hygiene and Tropical Medicine, London, United Kingdom; 4 Malaria Research and Training Centre, Faculty of Medicine, University of Bamako, Bamako, Mali; 5 Institut de Recherche en Sciences de la Santé, Direction Régionale de l'Ouést, Bobo-Dioulasso, Burkina Faso; 6 The Weatherall Institute of Molecular Medicine, University of Oxford, Oxford, United Kingdom; 7 Section of Parasitology, Department of Public Health Sciences, University of Rome La Sapienza, Rome, Italy; 8 Wellcome Trust Centre for Human Genetics, University of Oxford, Oxford, United Kingdom; Johns Hopkins University, United States of America

## Abstract

Highly parallel sequencing technologies permit cost-effective whole genome sequencing of hundreds of *Plasmodium* parasites. The ability to sequence clinical *Plasmodium* samples, extracted directly from patient blood without a culture step, presents a unique opportunity to sample the diversity of “natural” parasite populations in high resolution clinical and epidemiological studies. A major challenge to sequencing clinical *Plasmodium* samples is the abundance of human DNA, which may substantially reduce the yield of *Plasmodium* sequence. We tested a range of human white blood cell (WBC) depletion methods on *P. falciparum*-infected patient samples in search of a method displaying an optimal balance of WBC-removal efficacy, cost, simplicity, and applicability to low resource settings. In the first of a two-part study, combinations of three different WBC depletion methods were tested on 43 patient blood samples in Mali. A two-step combination of Lymphoprep plus Plasmodipur best fitted our requirements, although moderate variability was observed in human DNA quantity. This approach was further assessed in a larger sample of 76 patients from Burkina Faso. WBC-removal efficacy remained high (<30% human DNA in >70% samples) and lower variation was observed in human DNA quantities. In order to assess the *Plasmodium* sequence yield at different human DNA proportions, 59 samples with up to 60% human DNA contamination were sequenced on the Illumina Genome Analyzer platform. An average ∼40-fold coverage of the genome was observed per lane for samples with ≤30% human DNA. Even in low resource settings, using a simple two-step combination of Lymphoprep plus Plasmodipur, over 70% of clinical sample preparations should exhibit sufficiently low human DNA quantities to enable ∼40-fold sequence coverage of the *P. falciparum* genome using a single lane on the Illumina Genome Analyzer platform. This approach should greatly facilitate large-scale clinical and epidemiologic studies of *P. falciparum*.

## Introduction

Highly parallel sequencing technologies permit cost-effective whole genome sequencing of hundreds of *Plasmodium* parasites [1], [2] and promise to revolutionize our understanding of the global diversity and migration patterns of *Plasmodium* populations. This data will be essential to help monitor the spread of drug resistance and to facilitate the discovery of genomic regions under recent selection (e.g. anti-malarial drug pressure). It will also facilitate the identification of candidate targets for drug and vaccine development and enhance our knowledge of the basic biology of malaria parasites. The accessibility of whole genome sequencing platforms will provide the means to routinely perform large-scale clinical and epidemiologic studies on *Plasmodium*. In this context, simple but effective methods for processing clinical malaria samples, obtained directly from the patient blood without a culture-adaptation step (which is a costly and laborious approach, not always successful and subject to potentially selective pressures), are essential. These methods will present the opportunity for high-throughput investigations of the “natural” diversity in parasite populations, enable assessments of important *in vivo* phenotypes, and ensure that whole genome sequencing approaches are accessible to a wider spectrum of the malaria community including laboratories without parasite culture facilities.

A major challenge to sequencing clinical malaria samples using shotgun technologies is the abundance of “contaminating” human DNA. Owing to the random DNA fragmentation and sequencing approach of shotgun methods, all organisms present within a sample will be sequenced. Thus, if human DNA is abundant, this will greatly reduce the sequence coverage of the *Plasmodium* genome. Reduction of human DNA from clinical malaria samples is challenging owing to the abundance of human relative to *Plasmodium* DNA, even in high parasitaemia infections. In a whole blood sample with a 1% parasitaemia, the parasites may be ∼5–10 times more abundant than the white blood cells (WBCs). However, as the human genome is approximately 100-fold larger than the *Plasmodium* genome and is diploid, each WBC carries the equivalent DNA weight of about 200 early blood stage (haploid) *Plasmodium* parasites. At present, no effective method is available for separating human from *Plasmodium* DNA. However, a number of methods for reducing human DNA via separation of WBCs from the red blood cell (RBC) fraction have been described. These include the use of density-gradient separation of specific subsets of WBCs [3], [4], [5], [6], [7], [8], filter-based methods (including commercially available units and home-made cellulose filters) [5], [9], [10], [11], [12], [13], cell-sorting using flow cytometry technology [14], and magnetic-separation using commercial LD columns (MACS) [15] or magnetic dynabeads (Dynal) coated with anti-HLA1 antibody.

In the context of sample preparation for genome-wide *P. falciparum* shotgun sequencing, we sought a simple but effective method for removing WBCs from patient blood samples using standard laboratory facilities. In the first of a two-stage approach, a preliminary assessment of combinations of density-gradient separation, filtration and magnetic separation using anti-HLA1 dynabeads was undertaken on clinical malaria blood samples collected in Mali. The performance of each method was assessed for WBC-depletion efficacy and ease of use/labour intensity. This assessment indicated a two-step combination of Lymphoprep density-gradient centrifugation followed by Plasmodipur filtration as an efficient and simple method for removing WBCs. In the second stage of assessment, using a larger set of clinical malaria blood samples from Burkina Faso, we further validated our initial results on the two-step Lymphoprep/Plasmodipur process and we explored if other factors, such as sample storage and parasitaemia levels, could affect WBC depletion efficacy.

Depending upon the study objective and *Plasmodium* coverage requirement, varying levels of human DNA may be tolerated in shotgun sequencing. We assessed the influence of human DNA contamination on *P. falciparum* sequencing yield using genome-wide sequence data generated on the Illumina Genome Analyzer platform from a range of the clinical malaria samples described in the study.

## Materials and Methods

### Ethics

Ethical approval for the collection of patient samples from Mali and Burkina Faso was granted by the Comite d'Ethique de la Faculté de Médecine de Pharmacie et d'Odontostomatologie, Bamako, Mali, and Comite d'Ethique Institutionnel du Centre Muraz, Bobo-Dioulasso, Burkina Faso. All samples from adults were collected with informed, written consent from the patient. Samples from children were collected with informed, written consent from a parent or guardian.

### Sample collection

All samples were collected within the framework of a multicenter study using genome-wide SNP analysis to investigate the population genetic structure and diversity of *P. falciparum* samples from across the globe (Manske, Miotto et al., submitted). In both Mali and Burkina Faso, samples were collected in a clinical setting from patients of all ages with uncomplicated (according to the World Health Organization guidelines) falciparum malaria, as determined by microscopy. In Mali, samples were collected from Kolle and Faladje, two rural villages approximately 60 and 80 km, respectively from the Medical Research and Training Centre (MRTC) laboratories in Bamako. Transportation from either clinic to the MRTC, where WBC depletion processing was undertaken, involved a 2–3 hour journey by car over long stretches of unmaintained road. In Burkina Faso, samples were collected from three urban clinics in Bobo-Dioulasso (Colsamma, Ouezzin-ville and Sakaby), each up to 8 km from the laboratory at the Institute de Recherche en Sciences de la Santé (IRSS). Sample transportation from the clinics to the IRSS involved a 20–30 minute journey by moped over a mix of well and poorly maintained bitumen road. Samples were collected from consenting patients attending the clinic between October and November 2007 in Mali, and in September 2008 in Burkina Faso. In both studies, venepunctures were undertaken using 21 gauge butterfly needles (Becton Dickinson) and peripheral blood samples were drawn in EDTA-containing Vacutainer tubes (Becton Dickinson). Blood tubes were stored in cushioned cool boxes maintained at 4–8°C using cold packs. The following data was recorded for each sample: site, parasitaemia (and parasite density), blood volume, haemoglobin density, time and date of venepuncture, time and date of WBC-depletion processing, and method of WBC-depletion. All samples were collected with written, informed consent from a parent or guardian.

### WBC-depletion strategy

The following methods were each investigated in a minimum of 8 samples in the first stage of assessment; (a) Plasmodipur, (b) Lymphoprep followed by Plasmodipur, (c) Plasmodipur followed by anti-HLA1 dynabeads, and (d) Lymphoprep followed by anti-HLA1 dynabeads. Plasmodipur filtration was prioritized for assessment as it offered potentially the easiest and least time-consuming approach. Lymphoprep density-gradient centrifugation only separates the lymphocyte WBC fraction. However, in combination with other methods, this approach offered a cost-effective method for reducing the WBC fraction sufficiently to prevent potential saturation of downstream separation methods. Anti-HLA1 dynabeads were included as a final step to indiscriminately remove the WBCs remaining after prior depletion methods.

Amongst the methods not listed above, Plasmagel, which separates the granulocyte WBC fraction in a density-gradient manner, was excluded early on in the study as samples subject to this method failed to separate and formed a gelatinous composition leading to sample loss. Preliminary tests using homemade CF11 cellulose powder filters [5], [12] proved to be lengthy, and variation by operator and batch was observed. Amendments to the CF11 protocol are currently being undertaken to standardize column preparation and improve efficacy for *P. falciparum*-infected blood samples (Venkatesan *et al.*, in preparation). Flow cytometry systems were excluded from assessment owing to their inherent requirement for costly, specialized laboratory equipment. Purification using magnetic LD columns (MACS) was excluded as the system depends on the presence of haemozoin, which is absent from the early parasite stages which make up the majority of the population in peripheral blood samples. To overcome this problem, a short parasite maturation culture step is necessary prior to magnetic purification. Tests are currently being performed to verify the efficiency of this approach (Amaratunga and Fairhurst, in preparation).

### Lymphoprep density-gradient centrifugation

One blood volume of Lymphoprep (Axis-shield) was added to a 15 ml centrifuge. Blood samples were diluted with an equal volume of RPMI-1640 solution (Sigma) and carefully layered over the Lymphoprep, taking care to avoid mixing. It should be noted that other isotonic solutions can replace RPMI in this protocol (such as Phosphate Buffered Saline - PBS). A maximum of 5 ml diluted blood over 2.5 ml Lymphoprep was used per tube (larger volume samples were split accordingly). Samples were centrifuged at 800 g for 20 min at 21–22°C. The mononuclear cells, which formed a distinct band at the sample/medium interface were aspirated and disposed. The RBC pellet was washed with 10 ml RPMI and centrifuged at 800 g for 5 min. The supernatant was aspirated and disposed and the pellet was subject to further processing with anti-HLA1 dynabeads or Plasmodipur filters.

### Plasmodipur filtration

Plasmodipur filters (Euro-diagnostica) were pre-wet with 5 ml RPMI. Whole blood samples were diluted with 1 volume of RPMI, and post-Lymphoprep RBC pellets were diluted with 3 volumes of RPMI. The diluted blood was gently passed through the Plasmodipur filter using a syringe and collected in a 50 ml falcon tube. RBCs remaining in the filter were washed through with 10–20 mls RPMI. The filtered blood was centrifuged at 800 g for 10 min. The supernatant was removed, and the RBC pellet was either further processed with anti-HLA1 dynabeads or stored at -20°C until DNA extraction.

### Anti-HLA1 dynabead separation

Ten microliter aliquots of Dynal CELLection Pan Mouse IgG dynabeads (Dynal) were used per 5 ml RBC pellet. Dynabead aliquots were prepared by washing twice with 1 ml of PBS plus 1% bovine serum albumin (BSA), followed by resuspension in 390 ul PBS plus 1% BSA. Five microliters W6–32 mouse monoclonal antibody to human HLA 1 (Abcam) was added to each dynabead aliquot and incubated on a rotating wheel for 60 minutes at 22°C. After incubation, the W6–32 antibody-bound dynabeads were washed once in 1 ml PBS plus 1% BSA, and resuspended in 200 ul PBS and 1% BSA. Post-Lymphoprep/Plasmodipur RBC pellets were resuspended in an equal volume of RPMI-1640, added to the antibody-dynabead preparation and incubated on a rotating wheel for 40 minutes at 22°C. Post-incubation, the tubes were placed in a magnetic rack for 5 minutes, allowing the WBC-bound magnetic dynabeads to bind to the magnetic face of the tube. The supernatant was carefully removed without disturbing the dynabeads, transferred to a clean tube and washed twice by centrifugation at 800 g for 5 minutes at 22°C with RPMI-1460 medium.

### DNA extraction and Quantification

DNA extraction was undertaken using the QIAamp DNA Blood Midi/Maxi Kit (Qiagen) as per manufacturer's protocol. In order to quantify the abundance of human and *P. falciparum* DNA remaining after WBC-depletion, quantitative real-time PCR (QRT-PCR) analysis was undertaken on 2–5 ng aliquots of each sample using the Applied Biosystems StepOne PCR system with human (primers for TLR9 gene: ACGTTGGATGCAAAGGGCTGGCTGTTGTAG and ACGTTGGATGTCTACCACGAGCACTCATTC) and *Plasmodium*-specific (primers for EBA175 gene: ACGTTGGATGCACCAGTGAAGAAACTACAG and ACGTTGGATGCTTCATATTCCTTAGTAAGCG) primer sets. Human and *P. falciparum* DNA negative controls were included in the respective reactions, to confirm primer species-specificity. Each primer set was unique within the respective genomes. Pure human and pure *P. falciparum* standards were prepared to range 0.1–100 ng/ul concentrations. Test samples were diluted as necessary to fit within this range. All samples and standards were tested in duplicate. DNA (1 ul) was added to a 24 ul PCR solution containing 8.5 ul water, 12.5 ul PCR Sybergreen Master Mix and 1.5 ul of each primer (stock at 2 uM). The mixture was amplified for 5 cycles of 94°C for 45 seconds (sec), 56°C for 45 sec, 72°C for 45 sec, followed by 30 cycles of 94°C for 45 sec, 65°C for 45 sec (data collection point) and 72°C for 45 sec. Data was analyzed using the Applied Biosystems StepOne V2.0 software.

### Statistical Analysis

A multivariate analysis of variance (linear regression) model was used to a) measure the relative contributions of different factors to the observed variation in the percentage of human DNA and b) the relative contributions of human DNA level and sequence read length to variation in *P. falciparum* sequence coverage. The following variables were analysed: parasitaemia, study site, blood storage duration (from venepuncture to WBC-depletion), blood volume, haemoglobin density and WBC depletion method. The percentage of human DNA per sample was transformed using a square-root transformation to improve symmetry. Similarly, percent parasitaemia was logarithmic transformed and average genome coverage was transformed using a square-root. All analysis was performed using the R statistical package [16].

### DNA Sequencing

Fifty-nine samples with human DNA levels up to 60% (and a minimum of 500 ng total DNA) were sequenced on the Illumina Genome Analyzer platform (http://www.illumina.com/systems/genome_Analyzer.ilmn). Library preparation and sequencing was undertaken by the Wellcome Trust Sanger Institute core library preparation and sequencing teams. Standard paired-end libraries of 200–400 bp DNA fragments were prepared from 500–1000 ng total DNA. Sequence reads were either 54 bp or 76 bp long. Owing to continual improvements in read length, samples sequenced later in time had 76 bp sequence reads. Up to 3 lanes were sequenced per sample. Sequence data was mapped to the reference *P. falciparum* genome (3D7 version 4.1.2) using the SNP-o-matic read mapping tool [17] under the parameters for perfect read matching (with allowance for pre-identified SNPs) (Manske *et al.*, in preparation). Only sequences which mapped uniquely within the *P. falciparum* reference genome and which did not map to the human reference genome were included in our analyses. Coverage (average number of reads representing a given nucleotide) statistics on the number of bases mapped to the reference genome were recorded per lane for each sample.

## Results

### WBC-depletion assessment in Mali

**Figure 1:**
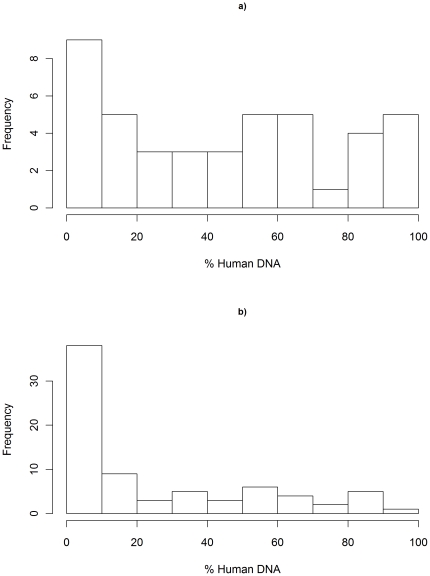
Frequency distribution of human DNA proportions post WBC-depletion. a) Mali, b) Burkina Faso. Human DNA proportions are presented as the percentage human DNA in the total sample as estimated by quantitative real-time PCR (see methods). The Malian samples (a) represent a range of WBC-depletion methods. All Burkinabe samples (b) were processed using the Lymphoprep plus Plasmodipur approach.

**Figure 2:**
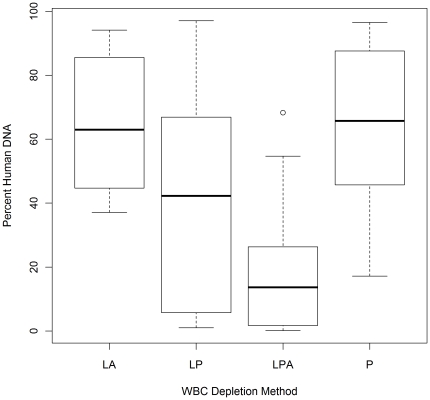
Distributions of human DNA proportions (% of total DNA) remaining after processing clinical blood samples from Mali with four different WBC-depletion methods. LA = Lymphoprep plus Antibody (N = 8), P = Plasmodipur (N = 13), LP = Lymphoprep plus Plasmodipur (N = 9), LPA = Lymphoprep plus Plasmodipur plus Anti-HLA1 (N = 13).

Forty-three samples were collected from the villages of Kolle and Faladje (24 and 19 respectively) and were processed to deplete WBCs using different approaches. A wide range of human DNA (Figure 1a) was observed after WBC-depletion. The samples processed with the Lymphoprep plus Plasmodipur plus anti-HLA1 approach exhibited the lowest% human DNA (median 13.6%, Interquartile range (IQR) 1.7–25.4), followed by Lymphoprep plus Plasmodipur (median 42.2%, IQR 5.7–66.9) (Figure 2). The Plasmodipur alone and the Lymphoprep plus anti-HLA1 approaches exhibited similar levels of human DNA (median 63.4%, IQR 45.7–87.6 and median 62.91%, IQR 44.6–85.6 respectively).

**Figure 3:**
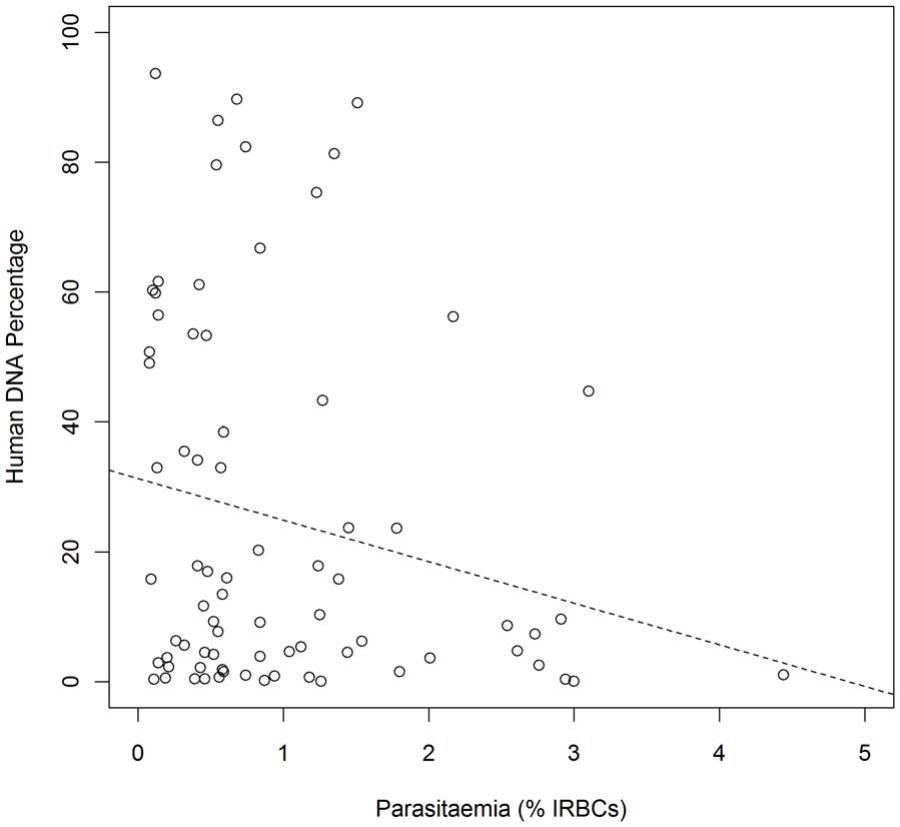
Human DNA percentage post-purification with Lymphoprep plus Plasmodipur against parasitaemia in Burkina Faso. The dashed line indicates the line of best fit. IRBC = Infected Red Blood Cell.

Moderate inter-sample variation was observed in a number of variables such as parasitaemia, (median 1.4%, range 0.7–9.9), blood volume (median 4.0 ml, range 2.0–8.0), haemoglobin density (median 10.4 g/dl, range 7.7–13.5) and duration of blood storage (median 4.5 h, range 2.2–102.0). These variables, study site and WBC-depletion method, were all fitted into a multivariate analysis of variance (linear regression) model to measure their relative contributions to the observed variation in the percentage of human DNA. The most significant determinant of the human DNA yield was the WBC depletion method used (F[3, 39] = 8.45, p = 0.00019). The only other variable which demonstrated a significant influence was parasitaemia (F[1,43]) = 4.32, p = 0.043). The multiple linear regression analysis (F[4,38] = 8.06, P = 0.000083) indicated that relative to the other approaches, the 3-method approach (Lymphoprep plus Plasmodipur plus anti-HLA1) was the most effective at reducing the percentage of human DNA (vs Lymphoprep plus anti-HLA1: coef = 4.45, se = 1.08 P = 0.0002; vs Plasmodipur: coef = 4.03, se = 0.90, P = 0.000064; vs Lymphoprep plus Plasmodipur coef = 2.32, se = 1.01, P = 0.027). High parasitaemia levels (coef = −3.34 se = 1.56, P = 0.039) also significantly reduced the percentage of human DNA. However, as illustrated in Figure 3, a large amount of variation determined by other unknown variables is observed in the correlation between parasitaemia and the percentage of human DNA. Thus, specific threshold parasitaemias for yielding human DNA percentages below a required threshold cannot be assigned.

Although the Lymphoprep plus Plasmodipur plus anti-HLA1 approach was most efficient at removing WBCs, this was also the most laborious approach. In particular, the antibody stage was especially lengthy owing to multiple washing steps and two long incubation periods. The simplest and least laborious of all the procedures was the Plasmodipur filtration. With regards to cost, Plasmodipur filters and antibody/dynabeads both approximate £10–15 per sample, while the Lymphoprep step is vastly cheaper at approximately £0.14 per sample. The Lymphoprep step also enables the collection of the lymphocyte fraction for other, human genetic or immunological, studies. Thus, the three-step method may be the best option for studies with small sample size or highly critical samples, while the Lymphoprep and Plasmodipur approach was the most time- and cost-effective, and was therefore selected for the consecutive studies in Burkina Faso.

### Assessment of Lymphoprep plus Plasmodipur in Burkina Faso

Seventy-six samples were collected, 30 from Colsamma, 17 from Ouezzin-ville and 29 from Sakaby. All samples were processed using the Lymphoprep plus Plamodipur approach. Relative to the Malian study, the distribution of human DNA levels was more skewed toward a lower percentage of human DNA (Figure 1b). Whilst the average human DNA in Mali was 46% (sd = 36.0), in Burkina Faso an average of 25% (sd = 28.4) was observed. Despite the eight-fold increase in sample size, the Burkina Faso dataset still demonstrated noticeable inter-sample variability in the presence of human DNA. Inter-sample variability was also observed for a number of variables: parasitaemia, (median 0.59%, range 0.12–4.44) blood volume (median 3.5 ml, range 2.0–8.0), haemoglobin density (median 11.0 g/dl, range 7.3–16) and duration of blood storage (median 3.0 h, range 1.1–8.3). These variables were fitted in a multivariate analysis of variance model to assess their contribution to the observed variation in the percentage of human DNA. Parasitaemia was the only variable that influenced the level of human DNA after WBC depletion (F[1,74] = 3.852, P = 0.044).

### Influence of Human DNA on *Plasmodium* Genome Sequencing Coverage

**Figure 4:**
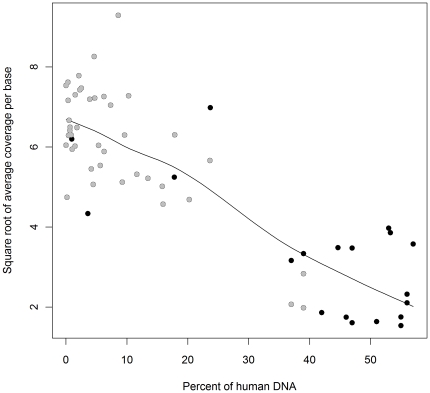
Square root of average coverage per base against human DNA proportion for 59 sequenced clinical samples. Sequence read lengths were either 54 bp (grey spots) or 76 bp (black spots). Each dot represents a sample. Where a sample was sequenced on more than one lane, the average sequence coverage is presented. The median (Square root of average coverage) intra-sample standard deviation was 0.91, and inter-quartile range was 0.70–1.35.

To assess the influence of human DNA on the sequencing coverage of the *Plasmodium* genome, we analysed the data available for 59 independent samples against the percentage of human DNA present in each sample. Sequence read length presented a potential confounder in this analysis as increased read length results in increased overall data yield and enhances read mapping ability. Furthermore, owing to these features, samples with moderately high levels of human DNA (>30%) were generally only sequenced once longer (76 bp) read lengths were available. We fitted a multiple linear regression model to assess the relationship between the average genome coverage per sequenced lane and the human DNA, whilst accounting for read length (54 or 76 bp). Percent of human DNA remained significant after adjusting for variability in read length (coef = −0.107, se = 0.015, P<0.0001). At both read lengths, a trend of increasing sequencing coverage with decreasing human DNA was observed (Figure 4). A summary of the sequencing data coverage for the 59 samples grouped by human DNA range and sequence read length is presented in Table 1. The highest average coverage per sequenced lane was observed for samples with ≤30% human DNA (median = 39.66, IQR 29.68–51.73). Higher human DNA levels resulted in lower average genome coverage (median = 4.90, IQR 3.15–11.82). With regard to the uniformity of sequence distribution across the *P. falciparum* genome, by sequencing one lane it was possible to obtain an average of 60% of the genome covered by at least 10 sequenced bases (minimum read depth 10), in samples with ≤30% human DNA. This rises to 70% and 85% average genome coverage when the threshold is reduced to at least 5 or 1 base respectively (minimum read depth of 5 or 1). For samples with >30% human DNA, sequence data from one lane yields an average of 21% of the genome being covered by at least 10 sequenced bases, rising to 32% and 60% for minimum read depth of 5 and 1, respectively.

**Table 1:** Summary of sequencing coverage per lane across 59 clinical samples.

% Human	Read Length	No. Samples (No. Lanes)	Average No. *P. falciparum* Megabases Sequenced	Average Coverage	% Genome Covered: Min Read Depth 1	% Genome Covered: Min Read Depth 5	% Genome Covered: Min Read Depth 10
<1	54	10 (18)	1006	43.19	91.11	75.26	64.78
	76	1 (2)	895	38.39	63.5	48.63	42.86
1–5	54	12 (20)	924	47.16	92.42	80.39	67.15
	76	1 (2)	437	18.8	65.67	47.16	37.17
5–10	54	8 (13)	1037	44.54	92.99	77.82	68.82
10–20	54	6 (11)	754	32.36	81.29	61.93	51.45
	76	1 (2)	641	27.5	88.7	71.32	58.95
20–30	54	2 (4)	629	26.98	92.52	73.7	60.51
	76	1 (2)	1136	48.74	96.44	86.87	80.22
30–40	54	3 (6)	126	5.4	54.01	21.91	12.58
	76	2 (2)	246	10.56	61.82	36.18	24.59
40–50	76	7 (13)	186	6.65	66.4	39.07	25.25
50–60	76	5 (11)	155	6.93	56.88	30.9	21.6

Data is averaged across all sample lanes within the given ranges of human DNA quantities. On average, each sample was sequenced on 2 lanes.

## Discussion

Highly parallel sequencing technologies such as Illumina's Genome Analyzer offer a cost-effective method to undertake genome-wide analysis on hundreds of *Plasmodium* samples. With ongoing technological developments, sample sizes in the order of thousands are anticipated in the near future. At this level, large-scale population genetic analyses of *Plasmodium* parasites using whole genome sequencing platforms should become commonplace. The use of clinical samples offer several advantageous features over cultured samples, including reduced time and cost of preparation, and, importantly, greater maintenance of the full diversity of parasites present in the original patient infection. However, owing to the indiscriminate nature of shotgun sequencing approaches, with current Illumina sequencing parameters, if a large quantity of the human WBCs are not removed from clinical samples prior to extraction, human sequence will be highly represented and *Plasmodium* sequence yield may be insufficient for proposed analyses.

We tested four different methods for removing WBCs from whole blood in a set of 43 samples collected at two health centres in Mali. The multiple linear regression coefficients indicated that relative to the other approaches, a combination of Lymphoprep plus Plasmodipur plus anti-HLA1 was the most effective at reducing the percentage of human DNA. However, this three-step approach also proved the most laborious. On this basis, and the approximate 2-fold increase in the cost of this three-step approach relative to the two-step Lymphoprep plus Plasmodipur approach, the latter was selected for the purification of a large set of samples from Burkina Faso intended for genome-wide sequencing using the Illumina Genome Analyzer platform.

Using just the Lymphoprep plus Plasmodipur method, the percentage of human DNA was lower in Burkina Faso than in Mali. In both studies, parasitaemia demonstrated a significant association with human DNA percentage, whereby increased parasitaemia correlated with reduced human DNA. No significant associations were observed with any of the other variables tested. In addition to parasitaemia, inter-sample variation in percent human DNA following Lymphoprep plus Plasmodipur processing may arise from a range of other variables not tested here. Physical variation between Plasmodipur filter units (e.g. possible air pockets), variation in the filtration procedure (e.g. force exerted on the membrane), variation in the Lymphoprep procedure (e.g. variable degree of mixing between blood and Lymphoprep layers), inter-individual variation in blood properties such as WBC count, variation in the venepuncture procedure, and continual growth of parasite populations between venepuncture and WBC processing may all contribute to the remaining inter-sample variability observed in human DNA levels. These factors should be further investigated in future studies to facilitate optimisation of the WBC depletion methods.

Depending upon the yield of *Plasmodium* sequence required for a given analytical procedure, varying levels of human DNA may be tolerated in shotgun sequencing. We assessed the *Plasmodium* sequence yield in 59 samples with human DNA levels up to 60%. As expected, human DNA contamination level influenced the yield of *Plasmodium* coverage even when accounting for read length. Lower human DNA levels will result in greater relative representation of *Plasmodium* DNA and, thus, sequence yield, which influences coverage levels. Amongst other variables, moderate variation in the total yield of *Plasmodium* sequence data between samples with the same human DNA levels and read length may arise as a result of technical inter-lane variation in DNA abundance on the flow cell (cluster density). Further details beyond the scope of this study on the sequence data presented here, including SNP detection approaches and descriptions of inter- and intra-host diversity, are presented elsewhere (Manske, Miotto et al., submitted). The data is also available in the European Nucleotide Archive (http://www.ebi.ac.uk/ena/data/search?query=plasmodium).

Using the Burkina Faso study as a model, at the 30% human DNA threshold and with 76 bp reads, using the Lymphoprep plus Plasmodipur approach, >70% of samples should yield average genome coverage of ∼40 with one lane of Illumina sequence data. Furthermore, as Illumina technology continues to improve, increasing sequence data yields are observed. In this study, 1–2 Gb total sequence data was generated per lane, whilst more recent lanes have produced yields of 4–5 Gb sequence data, a 2–3-fold increase in the average sequence coverage of the parasite genome despite consistent trends in the relative reduction of parasite sequence coverage with increasing human DNA (data not shown).

A number of WBC-depletion methods have been described in the malaria literature, but the majority of these studies have been concerned with removing WBCs to sufficiently low levels for parasite culture-adaptation. The efficacy of these methods in the preparation of clinical blood samples for whole genome sequencing has generally not been assessed. Addressing this in our study, we demonstrate that a simple two-step Lymphoprep plus Plasmodipur approach can be undertaken with basic laboratory facilities and is effective at removing human WBCs from clinical samples for large, genome-wide studies. For more bespoke studies on clinical samples, a combination of lymphoprep, Plasmodipur and anti-HLA1 dynabeads is more effective. A recent study demonstrated a yield of ∼50% human DNA following CF11-based filtration of a *P. vivax* sample with a 1.9% parasitaemia (Dharia et al., 2010). The yield of *P. vivax* Illumina sequence data (average 30-fold genome coverage) in this sample proved sufficient for genome-wide SNP-based analysis. Further analysis of the CF11 method with a larger sample size and broad range of parasitaemias should enable further validation of the utility of this method to *Plasmodium* sample preparation for whole genome sequencing. The observation of high sequence yields, even in samples with human DNA levels as high as 30 or 50%, validates the general approach of genome-wide sequencing on clinical *P. falciparum* samples and requests further optimization of cost-effective WBC-depletion methods for *P. falciparum* and other *Plasmodium* species. A combination of molecular (i.e. DNA quantity) and cellular (i.e. complete blood counts) assessments of human and parasite quantities both pre and post sample processing should facilitate these efforts.
